# High pretreatment plasma D-dimer predicts poor survival of colorectal cancer: insight from a meta-analysis of observational studies

**DOI:** 10.18632/oncotarget.20919

**Published:** 2017-09-15

**Authors:** Shao-Long Lu, Zhi-Hua Ye, Tong Ling, Si-Yuan Liang, Hui Li, Xiao-Zhun Tang, Yan-Song Xu, Wei-Zhong Tang

**Affiliations:** ^1^ Department of Colorectal Surgery, First Affiliated Hospital of Guangxi Medical University, Nanning, Guangxi Zhuang Autonomous Region 530021, P. R. China; ^2^ Department of Emergency, First Affiliated Hospital of Guangxi Medical University, Nanning, Guangxi Zhuang Autonomous Region 530021, P. R. China

**Keywords:** D-dimer, prognosis, colorectal cancer

## Abstract

D-dimer, one of the canonical markers of hypercoagulability, was reported to be a potential prognostic marker of colorectal cancer. However, an inconsistent conclusion existed in several published studies. Thus, we performed this meta-analysis to provide a comprehensive insight into the prognostic role for pretreatment D-dimer in colorectal cancer. Six databases (English: Pubmed, Embase and Web of Science; Chinese: CNKI, Wangfang and VIP) were utilized for the literature retrieval. Hazard ratio (HR) was pooled by Stata 12.0. A total of fifteen studies (2283 cases) corresponded to this meta-analysis and provided available data to evaluate the prognostic role of D-dimer for colorectal cancer. The pooled HR reached 2.167 (95%. CI (confidence interval): 1.672–2.809, *P* < 0.001) utilizing random effect model due to obvious heterogeneity among the included studies (I^2^: 73.3%; *P* < 0.001). To explore the heterogeneity among the studies, we conducted a sensitivity analysis and found a heterogeneous study. After removing it, the heterogeneity reduced substantially (I^2^: 0%; *P* = 0.549) and we obtained a more convincing result by fixed effect model (HR = 2.143, 95% CI = 1.922–2.390, *P* < 0.001, 14 studies with 2179 cases). In summary, high pretreatment plasma D-dimer predicts poor survival of colorectal cancer based on the current evidence. Further prospective researches are necessary to confirm the role of D-dimer in colorectal cancer.

## INTRODUCTION

Colorectal cancer, as a common malignancy in the world, has accounted for the second and third of cancer related death in male and female respectively [1]. Though the patients with early colorectal cancer reach satisfied survival, the advanced ones always own poor survival attributed to unresectable primary tumor, resistance and recurrence [2–4]. Thus, it is necessary to search for efficacious markers to assess the prognosis of colorectal cancer, especially in advanced stage.

Increasing evidence has observed aberrant blood coagulation in the patients with cancers [5, 6]. As an established risk factor of blood hypercoagulability, the tumor cells release various cytokines to activate coagulation [7, 8]. D-dimer is a canonical marker of hypercoagulability and a common approach to evaluate the hypercoagulable state in clinical practice [9]. Aberrant D-dimer has been detected in various cancers including colorectal cancer and several studies showed elevated D-dimer was correlated with poor survival of colorectal cancer [10, 11]. However, the inconsistent conclusion of pretreatment plasma D-dimer in colorectal cancer could not be ignored [12, 13]. Up to now, whether the pretreatment plasma D-dimer could be used for a predictive biomarker for the prognosis of colorectal cancer is controversial based on current evidence. Therefore, a comprehensive meta-analysis to combine the published studies is essential in order to reach a more convincing conclusion.

## RESULTS

### Literature retrieval

**Figure 1:**
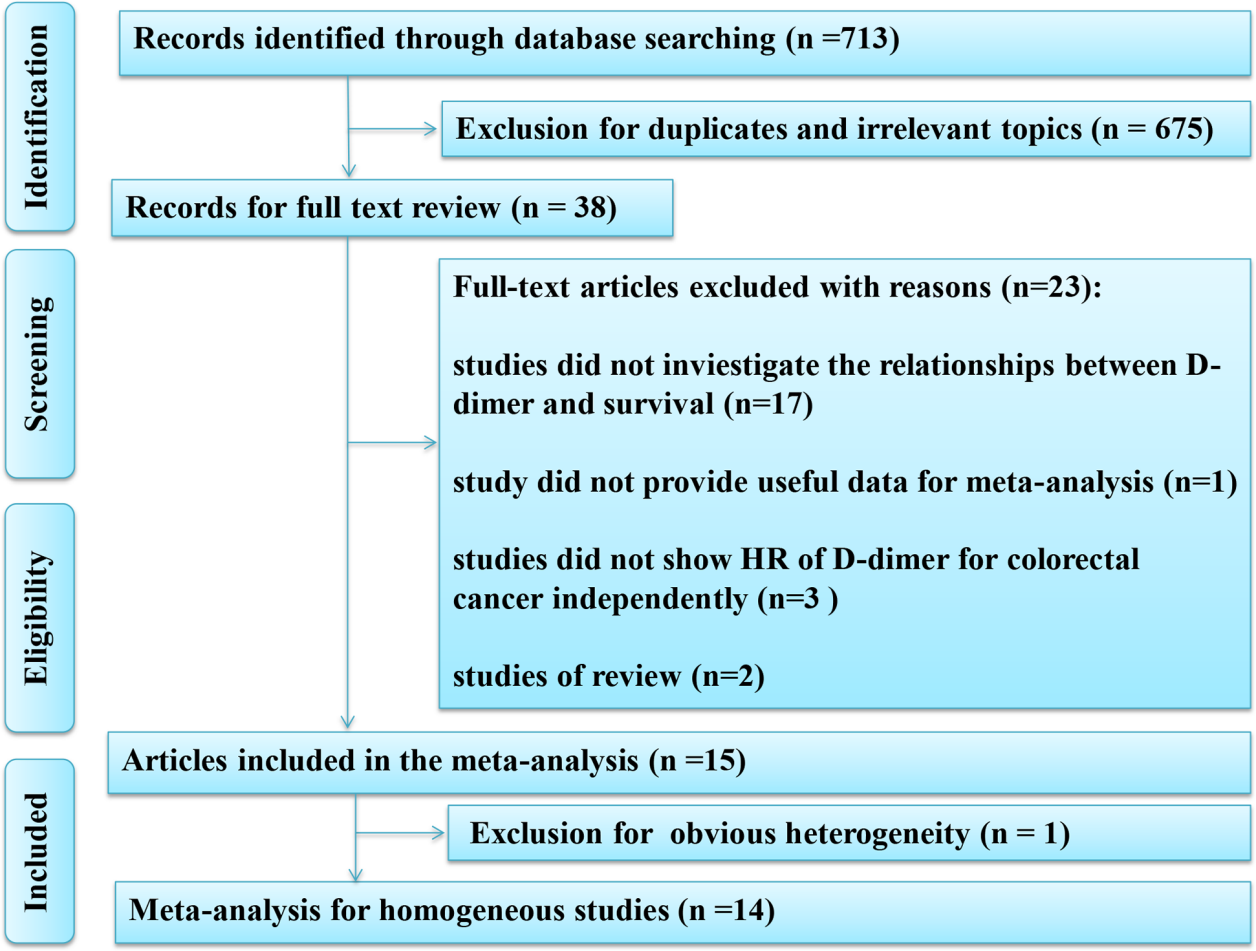
Flow diagram of the meta-analysis

A total of 713 records were identified from the initial search. Thirty-eight studies were analyzed with full-text after excluding the reduplicated or irrelevant studies. Finally, fifteen studies were used for this comprehensive meta-analysis and detailed information of eligible articles were presented in Table 1 [10–24]. The flow diagram of this meta-analysis was provided in Figure 1.

**Table 1:** The characteristics of the included studies

Author	Year	Region	Number of patients	Age (years)	Gender (Male/Female)	Tumor location (Colon/Rectum)	TNM stage	Dukes stage	Treatment	Cut-off value	Follow-up (months)	Survival analysis	HR	LL	UL	NOS score
Suee Lee	2017	South Korea	170	28-84(63^b^)	107/63	110/60	I-IV	NA	Surgery	1.4mg/L	>60	survival curve	5.890	2.050	16.950	7
Hong Tingting	2017	China	505	27-93(62.97^a^)	265/240	NA	I-IIIC	NA	Surgery	0.216mg/L	43*	multivariate	1.720	0.700	4.230	8
Kemal TekeGin	2016	Turkey	134	31-84(62.5^b^)	84/50	94/40	I-IV	NA	Surgery or RFA	0.96mg/L	18*	multivariate	1.873	1.032	3.494	8
Zhao Shuangshuang	2016	China	62	35-84	41/21	38/24	NA	NA	Chemotherapy	0.8mg/L	50	multivariate	2.38	1.082	5.235	8
Xu Haifei	2015	China	60	37-86(61.3^a^)	32/28	26/34	NA	A-D	Surgery	0.3mg/L	52.5*	multivariate	2.557	1.003	6.516	8
Ehsan Motavaf	2014	Denmark	166	38-94 (69^b^)	103/63	87/79	NA	NA	Surgery	0.3mg/L	60*	multivariate	2.200	1.100	4.800	8
Zhu Liming	2014	China	74	31-74(55.5^b^)	40/34	44/30	NA	NA	Chemotherapy	1.9mg/L	18.4*	multivariate	3.520	1.280	9.670	8
Xue Liying	2014	China	40	>=70(n=22); <70(n=18)	26/14	21/19	NA	NA	Chemotherapy	5.0mg/L	36	multivariate	4.386	2.101	9.174	8
Wang Junfeng	2013	China	341	23-90	178/163	188/153	I-III	NA	Surgery	0.5mg/L	64*	multivariate	1.829	0.110	7.893	8
Manabu Yamamoto	2012	Japan	42	>=70(n=30); <70(n=12)	26/16	25/17	NA	NA	Chemotherapy	1.0mg/L	14.1*	multivariate	3.749	1.127	18.160	8
Gurkan Tellioglu	2012	America	242	63^a^	81/161	NA	NA	NA	RFA	1.0mg/L	22*	multivariate	2.090	1.840	2.360	8
Corrado Pedrazzani	2010	Italy	199	26-94(67.7^b^)	118/81	141/58	NA	NA	Surgery	0.25mg/L	60	survival curve	1.500	0.860	2.610	8
Kilic M	2007	Turkey	51	29-80(60.9^a^)	34/17	NR	NA	A-D	Surgery	0.375mg/L	20*	survival curve	2.970	1.180	7.480	6
Kimberly Blackwell	2004	America	104	23-85(61^b^)	43/57	NA	NA	NA	Chemotherapy	0.001332mg/L	>25	multivariate	1.180	1.020	1.371	8
Masatoshi Oya	2001	Japan	93	27-84(62.7^a^)	62/31	51/42	I-III	NA	Surgery	0.85mg/L	54.7*	multivariate	1.874	1.001	3.497	7

a: mean age; b: median age; NA: not available; RFA: radio frequency ablation; *: median of follow-up; NOS: Newcastle-Ottawa Scale

### Association of elevated D-dimer and overall survival

**Figure 2:**
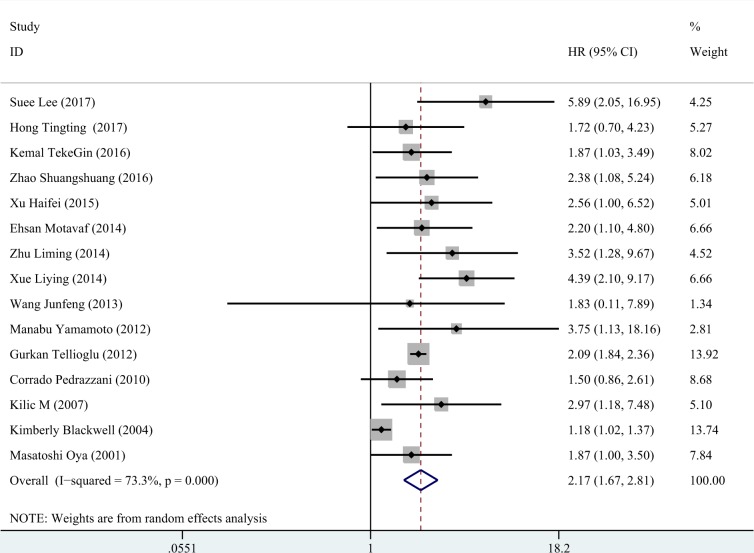
The forest plot of all included studies The pooled HR of all included fourteen studies was 2.167 (95% CI: 1.672-2.809, *P* < 0.001, random effect model due to obvious heterogeneity: I^2^ = 73.3%).

Fifteen eligible studies (a total of 2283 cases) which ranged from 2001 to 2017 included patients with colorectal cancer of TNM stage I-IV (Dukes stage A-D) that were accepted surgery and/or chemotherapy. Among the fifteen studies, twelve studies showed positive results of the relationships between elevated D-dimer and overall survival of colorectal cancer, and three studies obtained negative results. The combined HR of the fifteen eligible studies was 2.167 (95% CI = 1.672–2.809, *P* < 0.001, Figure 2) by a random effect model due to obvious heterogeneity (I^2^ = 73.3%, *P* < 0.001).

**Figure 3:**
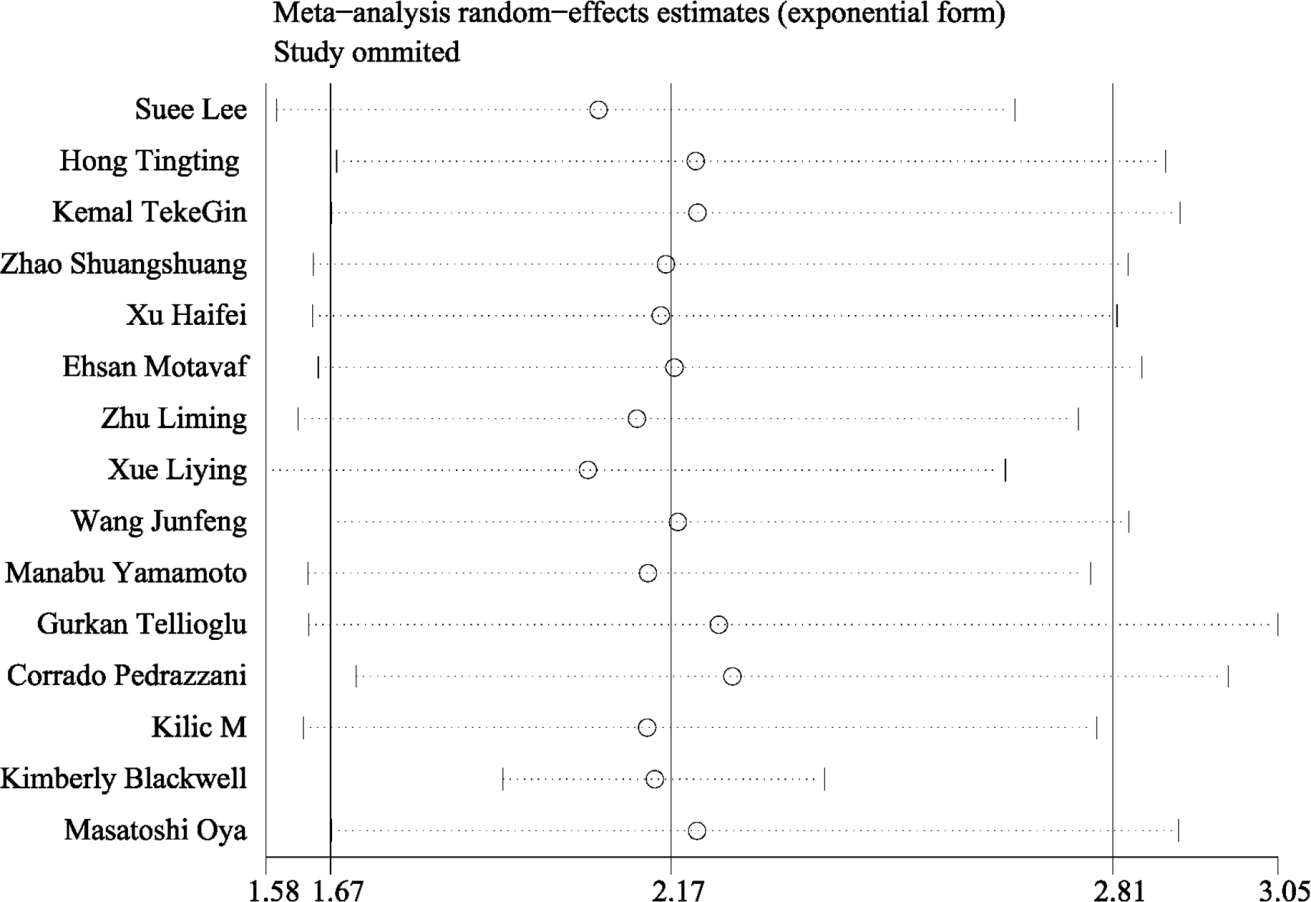
The sensitivity analysis of all included studies The combined result varied dramatically after excluding the study reported by Kimberly Blcakwell.

**Figure 4:**
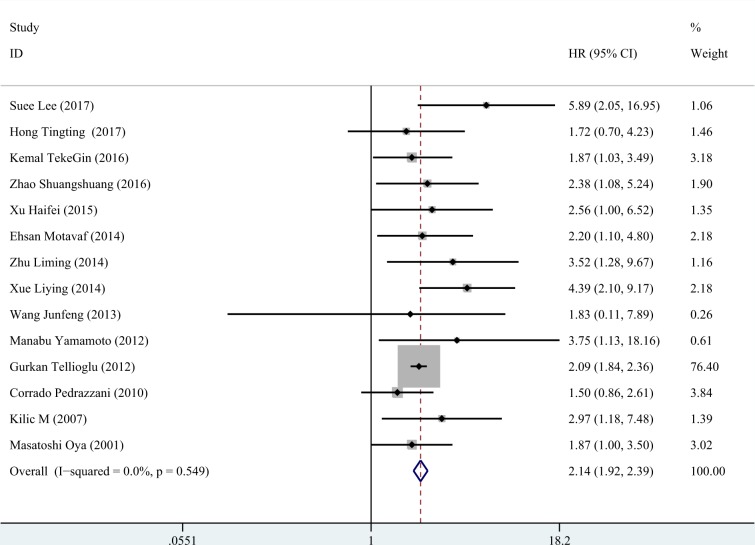
The forest plot of fourteen homogeneous studies The included homogeneous studies showed significant pooled HR of the prognostic value of D-dimer in colorectal cancer (HR = 2.143, 95% CI: 1.922–2.390, *P* < 0.001, fixed effect model due acceptable heterogeneity: I^2^=0%).

Subsequently, the sensitivity analysis was employed to investigate the source of heterogeneity among the eligible studies. We observed that the pooled result varied dramatically after removing a certain article (Kimberly et al.) with distinct cut off value of D-dimer (Figure 3). Therefore, we deleted it and gained a homogeneous pooled result (14 studies with 2179 cases) with fixed effect model which was more convincing (HR = 2.143, 95% CI = 1.922–2.390, *P* < 0.001; heterogeneity test: I^2^: 0%, *P*: 0.549, Figure 4)

### Subgroup analysis

**Figure 5:**
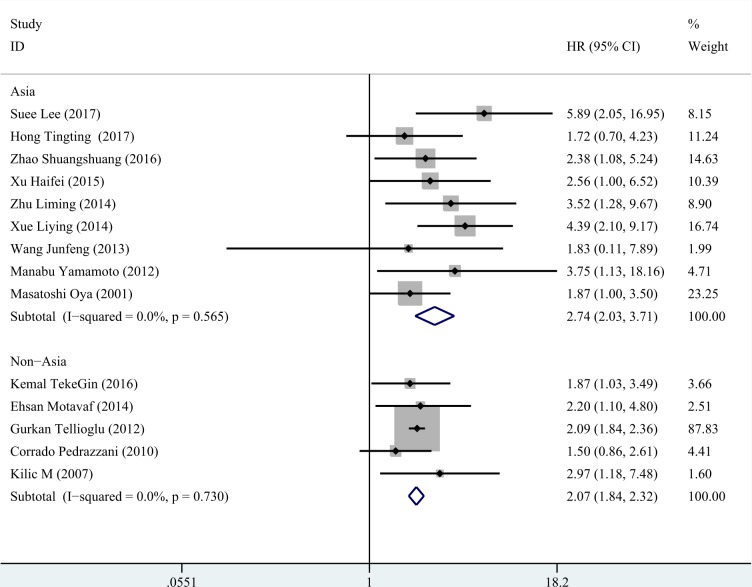
Subgroup analysis for region In Asia, the HR of D-dimer was 2.745 (95% CI: 2.030–3.711, *P* < 0.001, fixed effect model). In non- Asia, a similar result was observed (HR = 2.066, 95% CI:1.838–2.321, *P* < 0.001, fixed effect model).

**Figure 6:**
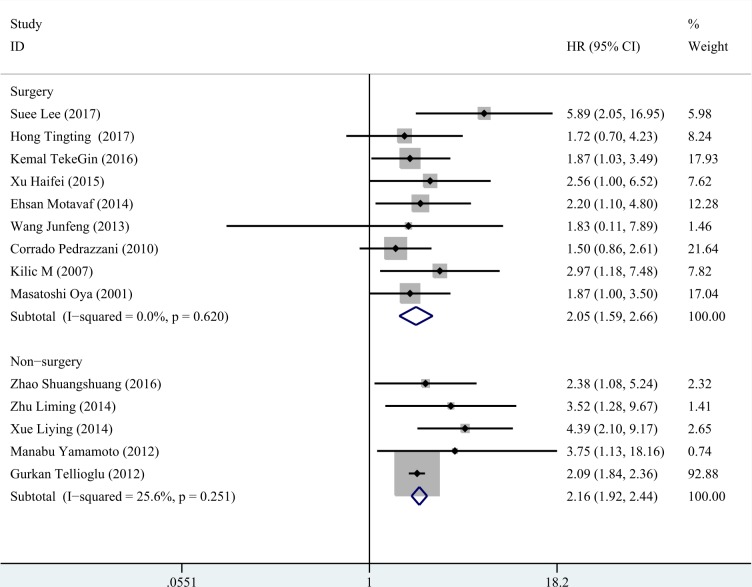
Subgroup analysis for treatment In the patients undergoing surgery, the HR of D-dimer was 2.055 (95% CI: 1.587–2.660, *P* < 0.001, fixed effect model) and a consistent result was gained in the patients received chemotherapy (HR=2.163, 95% CI: 1.919–2.439, *P* < 0.001, fixed effect model).

**Figure 7:**
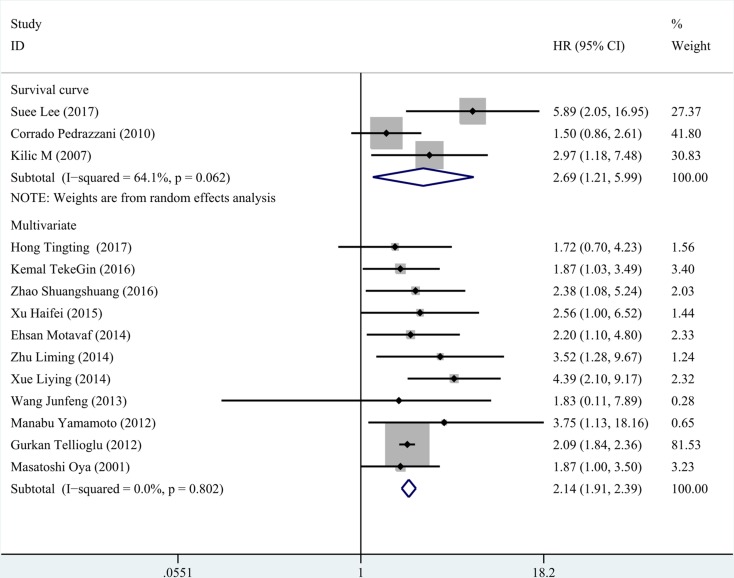
Subgroup analysis for the method of survival analysis The pooled HR from multivariate analysis was 2.140 (95% CI: 1.912–2.394, *P* < 0.001, fixed effect model) and pooled HR from survival curve was 2.692 (95% CI: 1.210–5.992, *P* = 0.016, random effect model).

We conducted subgroup analysis according to three factors (region, treatment and statistical method for survival). The results were presented in Table 2. Both in the studies of Asia and non- Asia, high D-dimer could predict poor survival of colorectal cancer, especially in Asia population (Figure 5). Likewise, we gained consist significant results in the rest two subgroup analyses (Figures 6, 7).

**Table 2:** Subgroup analysis of the meta-analysis

Subgroup	Number of studies	Pooled HR	95% CI		*P*	Medol	I^2^	*P*
Region								
Asia	9	2.745	2.030	3.711	< 0.001	Fixed effect	0.0%	0.565
non-Asia	5	2.066	1.838	2.321	< 0.001	Fixed effect	0.0%	0.730
Treatment								
Surgery	9	2.055	1.587	2.660	< 0.001	Fixed effect	0.0%	0.620
non-Surgery	5	2.163	1.919	2.439	< 0.001	Fixed effect	25.6%	0.251
Analysis of survival								
Multivariate	11	2.140	1.912	2.394	< 0.001	Fixed effect	0.0%	0.802
Survival curve	3	2.692	1.210	5.992	0.015	Random effect	64.1%	0.062

HR: hazard ratio.

### Publication bias

**Figure 8:**
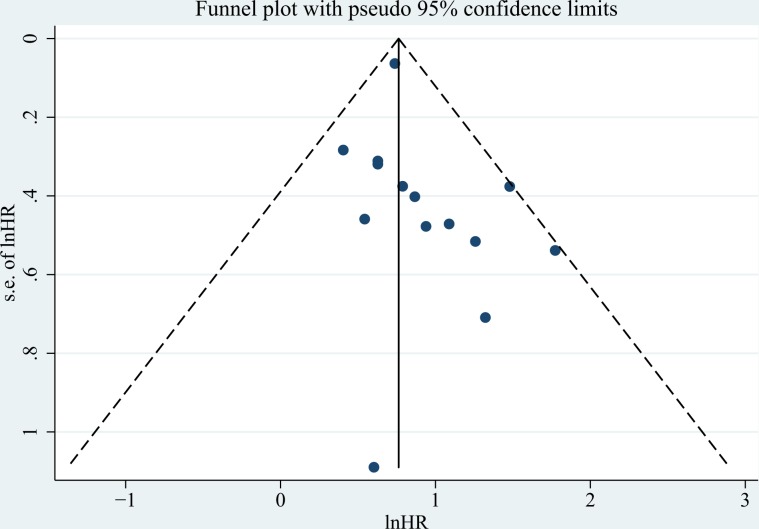
The funnel plot of included studies No obvious publication bias was observed (Egger's test: *P* = 0.153).

The funnel plot was presented in Figure 8 and no obvious was observed from the Egger's test (*P* = 0.292).

## DISCUSSION

Colorectal cancer, as a malignant neoplasm with high incidence worldwide, owns unsatisfied survival in advanced stage due to metastasis, recurrence and resistance of chemotherapy [1, 25, 26]. Thus, an efficacious biomarker to predict the prognosis of colorectal cancer is necessary, thereby providing potential target for treatment. D-dimer is a clinically common marker of activation of coagulation system. Increasing evidence showed that malignant neoplasm could promote the activation of coagulation, and elevated D-dimer was detected in several cancers which correlated to the prognosis, including in lung cancer, gastric cancer, and colorectal cancer [27–29]. Ma et al. have demonstrated high D-dimer predicted worse survival in lung cancer by a meta-analysis [30]. However, regarding colorectal cancer, the prognostic value of D-dimer was contentious based on the published studies.

In this current meta-analysis, we performed an integration of the current evidence (14 studies including 2179 cases) and provided a more stable and convincing result. The results of this current meta-analysis indicate high pretreatment plasma D-dimer could predict poor survival of colorectal cancer (HR = 2.143, 95% CI: 1.922–2.390). Subgroup analysis according to region, treatment and statistical method for survival, also showed consistent results: D-dimer could act as a predictive factor of survival both in the patients undergoing surgery, and the ones with metastasis received chemotherapy. The heterogeneity test and publication bias test all demonstrated the conclusion of the meta-analysis was stable.

In terms of the role of D-dimer in prognosis of patients with colorectal cancer, this following evidence may support our conclusion. At the genetic level, Vossen et al. confirmed that prothrombotic factor polymorphisms increased risk of colorectal cancer [31]. Yu et al. established that hypercoagulability owned a causal link to cancer-related genes (K-ras and p53) in colorectal cancer [32]. Meanwhile, the studies by Kemal et al., Wang et al. and Blackwell et al. revealed elevated D-dimer was associated with advanced *T* stage, positive lymph node metastasis, metastasis and cell differentiation [14–16]. Due to the close relationships between D-dimer and unfavorable clinicopathologic characteristics of colorectal cancer, the association between D-dimer and poor survival of colorectal cancer was understandable. Moreover, several studies clarified that plasma D-dimer of patents with colorectal cancer reduced obviously after surgery or chemotherapy which indicated the higher tumor burden the higher plasma D-dimer [33–35]. Therefore, the prognostic role of plasma D-dimer in pretreatment colorectal cancer was acceptable based on the aforementioned evidence.

Furthermore, the use of D-dimer for the prediction of survival in colorectal cancer should be based on a clear definition of high D-dimer because of the confounding factors, such as race, detection method, etc.

As Yu et al. reported, we could not assess D-dimer status exactly according to normal reference range in the patients with cancers [36]. Thus, tumor-specific D-dimer reference range should be further investigated with more epidemiological studies and provides a useful standard for clinical practice.

Though our study provided a more convincing conclusion that D-dimer could act as a predictive biomarker of prognosis for colorectal cancer, some inevitable limitations should be discussed: 1) several included studies did not show the TNM stage or Dukes stages of colorectal cancer and it may cause the heterogeneity among the included patients and affect the application of this meta-analysis; 2) a study [29] with negative result was excluded due to unavailable data for meta-analysis which may lead to an exaggerated positive conclusion; 3) limited sample size of several included articles may give a underpowered HR, and thereby impact the pooled HR; 4) studies only in English or Chinese were included which may lead to incomplete evidence collection. Thus, a prospective study with a large sample to confirm the conclusion of this meta-analysis and cover the above limitations is indispensable.

Collectively, our meta-analysis showed primary comprehensive insight into the significant role in prognosis of plasma D-dimer in pretreatment colorectal cancer and D-dimer maybe a potential target for the treatment of colorectal cancer. The patients with colorectal cancer may benefit from anticoagulation interaction. Moreover, further prospective investigations with large sample size are demanded to validate the role of D-dimer in colorectal cancer.

## MATERIALS AND METHODS

### Literature retrieval

In the present comprehensive meta-analysis, we searched six databases to collect the evidence, containing three databases in English (PubMed, Web of Science and Embase) and three databases in Chinese (database of China National Knowledge Infrastructure (CNKI), VIP and WanFang). The terms for retrieval were: 1) “D dimer” or D-dimer or “D-dimer fibrin” or “D-dimer fragments” or “fibrin fragment D1 dimer”; 2) colorectal or colon or rectal or bowel; 3) cancer or carcinoma or adenocarcinoma or tumour or tumor or malignanc* or neoplas*. The literature retrieval of this current meta-analysis was updated to June 12, 2017.

### Selection criteria

The eligible standards of the screen for the initial identified records were that 1) colorectal cancer should be pathological diagnosis, 2) detection of D-dimer was conducted in pretreatment colorectal cancer, 3) exploration of the relationships between D-dimer and prognosis of patients with colorectal cancer, and the data of prognosis was available directly or indirectly, 4) English or Chinese article, and 5) the recent study or study with a largest sample size will be included if the population is repetitive.

The exclusion criterias for the primary studies included 1) reviews, letters, conference data, and case reports, 2) an overlap among articles or duplicate data, 3) the use of animals, 4) unavailable HR. and 95% CI of the study

### Data extraction

Two authors screened the initial records independently and the final decision would be obtained by the third one if inconsistent conclusion existed. Information of the first authors, publication period, region, sample size, stage of colorectal cancer, cut off value, treatment, and HR were extracted. Moreover, we fellow a preferable order for HR extraction: HR (from multivariate analysis) > HR (from univariate analysis) > HR (extracted from Kaplan-Meier survival curve).

### Quality evaluation

Newcastle-Ottawa Scale (NOS) was employed to estimate the quality of each eligible studies [37]. A certain study is evaluated from 3 sections for the included cohort: selection, comparability and evaluation for outcome. The quality of a certain article was determined by stars summation of the above 3 sources and the study with star ≥ 5 is acceptable.

### Statistical analysis

The data combination of this meta-analysis was carried out by Stata 12.0. The prognostic value of plasma D-dimer in pretreatment colorectal cancer patients was evaluated via combined HR together with corresponding 95% CI. *Q* test as well as I^2^ statistic was utilized to estimate the heterogeneity of the pooled articles. In the meta-analysis, a model of fixed-effect was selected if heterogeneity of pooled studies was acceptable (*P* > 0.1 and I^2^ < 50%) [38]. If not, we used a model of random-effect to combine the HR. The publication bias was examined by Egger's test and *P* < 0.05 indicates statistical significance.
